# Study of Stem Cells Influence on Cardiac Cells Cultured with a Cyanide-P-Trifluoromethoxyphenylhydrazone in Organ-on-a-Chip System

**DOI:** 10.3390/bios11050131

**Published:** 2021-04-23

**Authors:** Anna Kobuszewska, Dominik Kolodziejek, Michal Wojasinski, Tomasz Ciach, Zbigniew Brzozka, Elzbieta Jastrzebska

**Affiliations:** 1 Faculty of Chemistry, Warsaw University of Technology, Noakowskiego 3, 00-664 Warsaw, Poland; akobuszewska@ch.pw.edu.pl (A.K.); dkolodziejek@ch.pw.edu.pl (D.K.); brzozka@ch.pw.edu.pl (Z.B.); 2 Department of Biotechnology and Bioprocess Engineering, Faculty of Chemical and Process Engineering, Warsaw University of Technology, Ludwika Waryńskiego 1, 00-645 Warsaw, Poland; Michal.Wojasinski@pw.edu.pl (M.W.); Tomasz.Ciach@pw.edu.pl (T.C.)

**Keywords:** heart-on-a-chip, cardiovascular diseases, stem cells, microfluidics

## Abstract

Regenerative medicine and stem cells could prove to be an effective solution to the problem of treating heart failure caused by ischemic heart disease. However, further studies on the understanding of the processes which occur during the regeneration of damaged tissue are needed. Microfluidic systems, which provide conditions similar to in vivo, could be useful tools for the development of new therapies using stem cells. We investigated how mesenchymal stem cells (MSCs) affect the metabolic activity of cardiac cells (rat cardiomyoblasts and human cardiomyocytes) incubated with a potent uncoupler of mitochondrial oxidative phosphorylation under microfluidic conditions. A cyanide p-trifluoromethoxyphenylhydrazone (FCCP) was used to mimic disfunctions of mitochondria of cardiac cells. The study was performed in a microfluidic system integrated with nanofiber mats made of poly-l-lactid acid (PLLA) or polyurethane (PU). The microsystem geometry allows four different cell cultures to be conducted under different conditions (which we called: normal, abnormal—as both a mono- and co-culture). Metabolic activity of the cells, based on the bioluminescence assay, was assessed in the culture’s performed in the microsystem. It was proved that stem cells increased metabolic activity of cardiac cells maintained with FCCP.

## 1. Introduction

Cardiovascular diseases (CVDs), with ischemic heart disease (IHD) at a forefront, are the leading cause of death worldwide [1]. Currently, heart transplantation is considered the most effective method of treating heart failure and improving the patient’s quality of life. However, the use of heart transplant has some limitations, such as: the risk of organ rejection and the small number of donors [2]. Regenerative medicine, which has been developing in recent years, might turn out to be one of the methods that will improve the effectiveness of heart failure treatment.

Regenerative medicine, as an interdisciplinary field, is focused on the repair, replacement of damaged cells, tissues, and organs in order to restore impaired function resulting from any cause (injury, birth defect, or disease) [3]. In developing new methods of treating cardiovascular diseases, research related to cell therapy and tissue engineering seem to be the most promising. Cell therapy involves direct transplantation to improve the functionality of the damaged organ. In turn, tissue engineering uses living cells, biocompatible materials, and biochemical and physical factors to create tissue-like structures that would repair or replace damaged tissue after transplantation [4]. Cells derived from donor tissue are used to form tissue in vitro. However, because of their limited number, the use of stem cells is becoming more popular. Stem cells are characterized by high proliferation capacity and the ability to differentiate into any type of cells [5].

Scientists are working intensively to differentiate stem cells into many types of cells, namely, cardiomyocytes, neurons, podocytes, osteoblasts, hepatocytes, epithelial cells, or adipocytes [6]. Because the heart is an organ with a low capacity for self-regeneration, research on the differentiation of stem cells towards cardiomyocytes is particularly important. It has been reported that only 1% of all cardiomyocytes are replaced by new cardiac cells each year. Consequently, spontaneous repair of the damaged cardiac tissue is not possible and results in heart failure [7,8]. For this reason, stem cells could prove to be an excellent alternative in treatment of heart failure. Embryonic stem cells (EMCs), induced pluripotent stem cells (iPSCs), and mesenchymal stem cells (MSCs) are most used for cardiomyocyte differentiation. Differentiation of these cells could be induced by biochemical, physical, or mechanical methods [7,9,10]. Cytokines (e.g., bone morphogenetic protein (BMP)), growth factors (e.g., transforming growth factor β (TGF- β)), fibroblast growth factor (FGF), and chemical reagents (e.g., 5-azacytidine, retinoic acid) are used in the biochemical methods [11,12,13,14,15]. In addition to biochemical methods, physical factors such as flexibility, stiffness, and topography of the cell culture material are increasingly used for the differentiation of stem cells. The use of an electric field is also used to differentiate stem cells into cardiomyocytes. This is an important factor because myocardial tissue is constantly subjected to electrical stimulation under physiological conditions [10]. Cardiac cells are also constantly exposed to various mechanical stimulations under native conditions, for example, force of muscle contraction or blood flow. Therefore, the effect of mechanical factors such as tensile and shear stress on stem cell differentiation is also investigated [16,17]. The extracellular microenvironment and co-culture with mature cells are also recognized as factors that could influence the fate of stem cells. The presence of mature cardiac cells and their physical contact with the stem cells increases the transduction of molecular signals leading to differentiation into cardiomyocytes [10]. Spontaneous beating and expression of specific cardiac marker (troponin) are commonly observed in stem cells that differentiated by contact with mature cardiac cells [18,19].

In vitro experiments are most often carried out on a macroscale using standard culture vessels (culture bottles, dishes, or multi-well plates) in which cell culture conditions differ from those which are present in a living organism [20]. Therefore, a tool is needed that could ensure appropriate culture conditions by combining the mechanical, biochemical, and physical signals that occur in the microenvironment of stem cells. Microfluidic systems called Organ-on-a-Chip could be a useful tool to create advanced models of cell cultures. So far, the use of microsystems for cell cultures has improved research in cell biology, disease modeling and development of new treatments [21]. The microsystems have many advantages such as: shortening the analysis time, reducing the consumption of reagents, mimicking dynamic (flow) conditions, and analyzing the influence of external factors in real time. The possibility of designing the geometry of microstructures in such a way that their dimensions correspond to the size of cells and resemble their natural microenvironment is an important feature of the microsystems [22,23]. It is also possible to integrate microsystems with biocompatible materials used for cell culture, for example, scaffolds, nanofibers, and hydrogels. Polymer scaffolds in the form of nanofibers are particularly important in research that uses cardiac cells. This is because the specific structure of nanofibers (their parallel arrangement) influences the direction of cell growth. Moreover, nanofiber mats are characterized by a high surface to volume ratio, high porosity, and an extracellular matrix-like structure that occurs in vivo [24]. So far, several experiments in which nanofiber mats were integrated with microsystems are described in the literature [25,26,27]. Nanofiber mats placed in the microsystems are mainly used as a substrate for cell culture [25]. The impact of nanofibers integrated with the microsystem on the arrangement and morphology of the MSCs and their fibrochondrogenic differentiation are described in other studies [26]. Hesari et al. conducted similar research. In the designed microsystem, the influence of nanofiber mats on the differentiation of iPSCs into neurons was investigated [27].

The aim of our research was to study the effect of stem cells on cardiac cells, in which disfunction of mitochondria was obtained. The studies were performed in the microfluidic system integrated with nanofibers mats used as a physical factor influences cell growth. There are some examples of the microsystems used for the culture and differentiation of stem cells [6,28,29], mimicking the disease state and co-culture stem cells with other cell types [30,31,32]. However, it is necessary to conduct research with the understanding of the processes that occur during the regeneration of damaged heart tissue. Contrary to other works, here we combine different factors (abnormal conditions, stem cell co-culture with cardiac cells, microfluidic conditions, nanofibers for mimicking parallel arrangement of the cells) which could help to study stem cells’ influence on the function of heart cells. The developed microfluidic system, integrated with nanofibrous mats, enabled four independent cell cultures to be conducted simultaneously under different conditions (normal, abnormal—as a mono- and co-culture). To the best of our knowledge, the effect of human mesenchymal stem cells on metabolically perturbed rat or human cardiac cells was evaluated for the first time.

## 2. Materials and Methods

### 2.1. PLLA and PU Nanofiber Mats

Poly-L-lactic acid (PLLA) (a mean fiber diameter of 186 ± 65 nm) and polyurethane (PU) (a mean fiber diameter of 318 ± 102 nm) nanofibrous mats produced using a solution blow spinning process were used in the research. The fabrication method and characterization of nanofiber mats were described in detail in our previous study [33,34]. Mats in the shape of discs (6 mm diameter) with cut-off sides creating a 4 mm width (alignment of fibers was parallel to the direction of the cut-off side) were prepared for integration with the designed microsystem.

### 2.2. Development of Organ-on-a-Chip System

**Figure 1:**
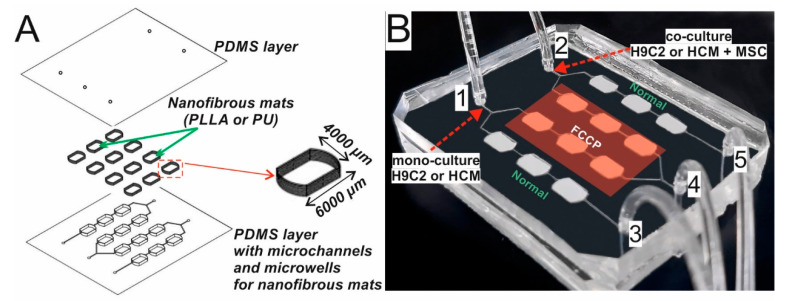
(**A**) A layout of the layers from which the microsystem is constructed. (**B**) The fabricated microsystem bonded with the second PDMS layer. Inlets are numbered: 1,2—inlets for cell suspension and outlets for reagents, 3,4,5—inlets for reagents and outlets for cell suspension (3,5—inlets for medium, 4—inlet for FCCP). PDMS–poly(dimethylsiloxane), PLLA-Poly-L-lactic acid, PU–polyurethane, FCCP-cyanide p-trifluoromethoxyphenylhydrazone, H9C2–rat cardiomyoblasts, HCM – human cardiac myocytes, MCS–mesenchymal stem cells.

The microstructures of the microsystem were fabricated using micromilling and replica molding methods. Basically, a mixture of prepolymer poly(dimethylsiloxane) (PDMS) and curing agent (mixed in 10:1 ratio) was poured onto a poly(methyl methacrylate) (PMMA) stamp made by micromilling. The stamp with PDMS mixture was cured for 19 min at 75 °C and the PDMS replica was peeled off the stamp. Next, nanofibrous mats were placed in each microwell and the PDMS layer was cured for the next 41 min [34]. The direction of the nanofibers’ arrangement in the mats was parallel to the direction of solution flow in the microsystem. The depth of the microwells (6400 µm length, 4000 µm width) depended on the thickness of the fabricated mats. It equaled 500 µm for PU mats and 350 µm for PLLA mats. Because of the microchannel network and nanofibrous mats placed in one PDMS layer, a smooth (bottom) PDMS layer was used to seal the microsystem. The bottom layer was obtained by mixing PDMS prepolymer and a curing agent (a ratio of 10:1) and cured for 1 h at 75 °C. The holes for inlets/outlets with a diameter of 1.3 mm were made in this layer. After that, both PDMS layers were bonded using surface plasma activation (Diener Electronic Atto). The fabricated microsystem is presented in Figure 1.

### 2.3. Flow Simulation in the Microsystem

Computer modeling simulations were performed using ANSYS software. The flow velocity distribution, shear stress, and the distribution of cell suspension were verified in the designed microsystem. In the case of flow and shear stress simulations, the flow rate of the culture medium introduced into the microsystem was 2 µL/min at external inlets (3 and 5) and 4 µL/min at internal inlet 4. While the simulation of the distributions of cell suspension in the culture microchambers was performed at a flow rate of 15 µL/min (for inlets 1 and 2). For the simulation of cell distribution in the microchambers, cell densities of 10^4^, 10^5^, 5 × 10^5^, and 10^6^ cells/mL were used.

### 2.4. Cell Lines

Rat cardiomyoblasts (H9C2) obtained from European Collection of Authenticated Cell Cultures, human cardiomyocytes (HCM) obtained from ScienCell Research Laboratories, and human mesenchymal stem cells (MSC) derived from Wharton’s jelly obtained from Polish Stem Cell Bank were selected for the study. The cells were cultured in DMEM high glucose (Biowest) (for H9C2 cells), DMEM F-12 (Gibco) (for HCM cells) and DMEM low glucose (Sigma-Aldrich) (for MSC cells) medium supplemented with 10% *v*/*v* FBS (Biowest), 1% *v*/*v* 100 mM penicillin-streptomycin (Biowest), and 1% *v*/*v* 25 mM L-glutamine (Biowest). HCM culture medium additionally contained 1% *v*/*v* 100 mM sodium pyruvate (Sigma-Aldrich), 0.01% *v*/*v* cardiac myocyte growth supplement (CMGS, ScienCell), and 0.01 % *v*/*v* MEM non-essential amino acids (NEAA, Sigma-Aldrich). All cell lines were cultured at 37 °C in a humidified 5% CO_2_ atmosphere.

### 2.5. Cell Culture in the Microsystem

A trypsinization of the cells cultured in a 25 cm^2^ culture flask was performed. For this purpose, cell cultures were washed with phosphate buffered saline (PBS, Biowest). Then, 0.25% Trypsin (Biowest) (for H9C2 and HCM cells) or TrypleExpress (Life Technologies) (for MSC cells) was added to the cells. After detaching, the cells were centrifuged at 1500 rpm for 5 min (for the H9C2 and HCM cells) or at 1780 rpm for 7 min (for the MSC cells). Finally, the pellet was suspended in a culture medium to obtain suspensions with a density of 10^4^, 5 × 10^5^, and 2.5 × 10^5^ cells/mL. Firstly, the microsystem was sterilized by the usage an ultraviolet (UV) light (Black Ray) for 30 min. Next, to provide a suitable microenvironment for the cell culture, the microsystem was filled with the culture medium and placed in an incubator at 37 °C and 5% CO_2_ overnight. After that, the prepared cardiac cell suspensions 5 × 10^5^ (through inlet 1) and 2.5 × 10^5^ cells/mL (through inlet 2) were introduced in the microsystem with a flow rate of 15 µL/min. Next, the microsystems were kept in an incubator overnight. For the test in a 96-well plate, 10^4^ cells were seeded in each well and placed in an incubator overnight. PU nanofibrous mats were used for culture of H9C2 cells, whereas PLLA nanofibrous mats were utilized for culture of HCM cells [33]. Therefore, the results for H9C2 and HCM cells, shown in figures, correspond to tests with PU and PLLA, respectively.

### 2.6. Cell Incubation with FCCP

A potent mitochondrial oxidative phosphorylation uncoupler—cyanide p-trifluoromethoxyphenylhydrazone (FCCP, Abcam) was used in the experiments. First, a stock solution of 20 mM FCCP in DMSO was prepared. Finally, 10 µM of the FCCP solution in a culture medium was used in the tests. The used culture medium was the same as for the routine culture of H9C2 and HCM cells, the difference being that DMEM without phenol red (Sigma-Aldrich) was used as a medium base. The solution of FCCP was introduced into the microsystem with the cultured cardiac cells (a flow rate of 2 µL/min for 15 min) through inlet 4. Then, the cells with FCCP were incubated at 37 °C and 5% CO_2_ for 30 min. At the same time, a fresh medium was introduced through inlets 3 and 5. Furthermore, 24 h later, a fresh medium and 2.5 × 10^5^ cells/mL of MSC cells were introduced through inlets 1 and 2, respectively. Finally, the microsystem was placed in an incubator for the next 24 h.

### 2.7. Evaluation of Changes in the Potential of the Inner Mitochondrial Membrane

To evaluate the changes in the mitochondrial membrane potential, which indicates that cells have entered the state of early apoptosis, the fluorescent cationic carbocyanine dye-5,5′,6,6′-tetrachloro-1,1′,3,3′-tetraethylbenzimidazolyl carbocyanine iodide (JC-1) (Biotium) was used. For this purpose, the dye solution at a concentration of 5 µM was prepared in a culture medium without phenol red immediately before the test. In the last day of the experiments performed in macroscale (using 96-well plate), the culture medium was removed from the cells and 100 µL of the JC-1 solution was added to each well. The cells were incubated with dye in an incubator (37 °C and 5% CO_2_) for 30 min. After this time, the dye solution was removed and 100 µL of a fresh culture medium without phenol red was added and the cells were incubated (37 °C and 5% CO_2_) for the next 60 min. After incubation, the intensity of fluorescence emitted for aggregates (λ_ex_ = 475 nm; λ_em_ = 590 nm) and monomers (λ_ex_ = 475 nm; λ_em_ = 530 nm) was measured using a multi-well plate reader (Cytation 3, BioTek). The results are presented as the aggregate to monomer ratio. Furthermore, 0 µM of FCCP (cardiac cells incubated with a culture medium) was used as a control. Additionally, an inverted fluorescence microscope (Olympus IX 71) was used to analyze the stained cells.

### 2.8. Evaluation of the Metabolic Activity of Cardiac and Stem Cells

AlamarBlue assay was used to assess the metabolic activity of the cells. Furthermore, 10% of AlamarBlue solution was prepared immediately before the experiments in a culture medium without phenol red. The tests were performed every culture day in macro- and microscale. In macroscale, the culture medium was removed from the 96-well plate, and 100 µL of 10% AlamarBlue solution was added. In microscale, the reagent was introduced into the microsystem through inlets 3, 5 (a flow rate of 2 µL/min), and inlet 4 (a flow rate of 4 µL/min) for 15 min. When the AlamarBlue solution was placed with the cells, the cultures were kept in an incubator (37 °C, 5% CO_2_) for 60 min. After that time, the fluorescence intensity was measured using a multi-well plate reader (Cytation 3, BioTek) (λ_ex_ = 552 nm; λ_em_ = 583 nm). After the measurement, the AlamarBlue solution was removed from the cells cultured in the 96-well plate and 100 µL of fresh culture medium was added. In the microsystem, a fresh culture medium was introduced into the microsystem through inlets 3, 4, and 5. Next, the cell cultures in macro- and microscale were continued. Furthermore, 0 µM of FCCP (cardiac cells with a culture medium) was used as a control.

To study the state of the cell cultures after 5 days, CellTiter-Glo 3D Cell Viability Assay (Promega) was used. For this purpose, 100 μL of CellTiter-Glo 3D reagent was mixed with 100 μL of DMEM without phenol red and the obtained solution was introduced into the microsystem for 15 min (through inlets 3, 5 with a flow rate of 2 µL/min, and inlet 4 with a flow rate of 4 µL/min). Then, the microsystem was incubated without light for 25 min at room temperature. After that time, the bioluminescence was determined using a multi-well plate reader (Cytation 3, BioTek). Furthermore, 0 µM of FCCP (cardiac cells with a culture medium) was used as a control.

### 2.9. Statistical Analysis

The experimental data were expressed as the mean ± standard deviation (SD) from at least three experiments. One-way analysis of variance (ANOVA) was used for statistical analysis. *p*-values of less than 0.05 were considered statistically significant (asterisk indicates *p* < 0.05).

## 3. Results

### 3.1. Initial Studies in Macroscale

Before starting the tests in the designed microsystem, preliminary studies were carried out in macroscale. This made it possible to assess the effect of stem cells on cardiac cells which were metabolically perturbed with high concentration of FCCP. For this purpose, a cell suspension of cardiac cells (H9C2 or HCM) with a density of 10^4^ cells/well was seeded into a standard 96-well plate. In turn, the cardiac cell suspension with a density of 5 × 10^3^ cells/well was seeded into the subsequent wells in which co-culture of cardiac cells with stem cells had been planned. A high concentration of FCCP (>5 µM) causes a significant mitochondrial depolarization and completes mitochondrial uncoupling [35,36,37]. There are some researches in which a high concentration of FCCP was also utilized to mimic a myocardial hypoxia environment in microchip [38]. Based on that and our previous results, 10 µM of FCCP was used to mimic abnormal conditions in the proposed study [34]. For this purpose, cardiac cells were metabolically perturbed by the addition of FCCP for 30 min. After this time, FCCP solution was replaced with a fresh culture medium. Then, MSC cells were added to the appropriate wells (the density of the suspension was 5 × 10^3^ cells/mL). AlamarBlue assay was performed daily on the following days of the culture to assess the condition of the cell culture. A culture of cardiac cells maintained in a culture medium was used as a control.

**Figure 2:**
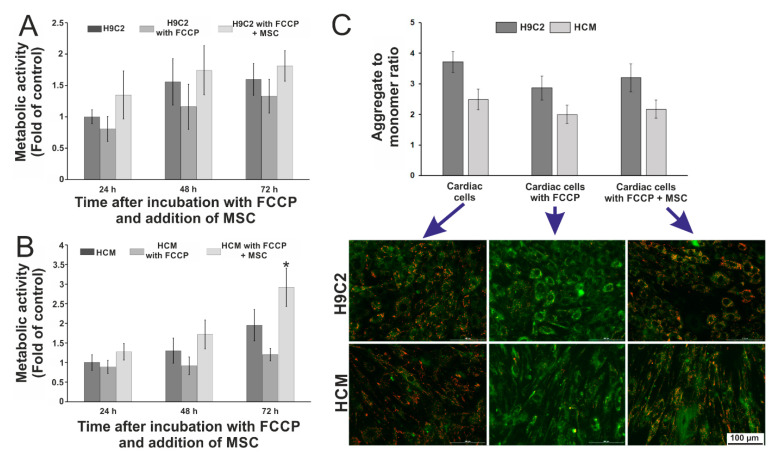
The results of metabolic activity measurements of (**A**) rat cardiomyoblasts (H9C2) and (**B**) human cardiomyocytes (HCM) after incubation with cyanide p-trifluoromethoxyphenylhydrazone (FCCP) and co-culture with mesenchymal stem cells (MSC). (**C**) Comparison of aggregate to monomer ratios for each tested cell culture type: cardiac cells cultured only in a culture medium, cardiac cells incubated with FCCP, and co-culture of cardiac cells incubated with FCCP with MSCs. The pictures of the cells stained with JC-1 dye (red—aggregates, green—monomers) are presented below. *N* ≥ 3, an asterisk indicates *p* < 0.05.

It was found that stem cells increased the metabolic activity of cardiac cells incubated with FCCP. In the case of H9C2 cells (Figure 2A) it increased daily in every type of culture. The lowest metabolic activity was observed for cardiac cells incubated with FCCP, whereas the highest value (two times higher compared to the control) of this parameter was noticed for H9C2 cells exposed to FCCP and co-cultured with stem cells. Moreover, this activity was slightly higher than the activity of cardiac cells cultured with a culture medium. Similar results were obtained for human cardiomyocytes HCM (Figure 2B). Metabolic activity increased for each type of culture in the following hours of the experiment. Similar to the H9C2 cells, HCM cells incubated with FCCP also exhibited low metabolic activity. It was found that for co-culture of HCM cells (exposed to FCCP) with MSCs, the measured parameter increased more than 1.5 times after 48 h and 3 times after 72 h of culture. This may indicate that stem cells have a stronger effect on HCM cells than on H9C2 cells maintained with FCCP.

Additionally, the changes in the potential of the mitochondrial membrane were examined on the last day of the cultures. For this purpose, JC-1 fluorescent dye was used. This dye exists in two forms: aggregates (a high potential) in living cells that emit orange/red fluorescence (λ_em_ = 590 nm) and monomers (a low potential) in apoptotic cells that emit green fluorescence (λ_em_ = 530 nm) [39,40,41,42]. It was assumed that stem cells could influence changes in the inner mitochondrial membrane potential. The results of the test are presented in Figure 2C. In the case of H9C2 cells, the aggregate to monomer ratio was lower for the cells treated with FCCP agent compared to the ratio of both dye forms for the cells incubated with a culture medium. A slightly higher value of this ratio was obtained for H9C2 cells incubated with FCCP and co-cultured with MSC cells. For human cardiomyocytes, aggregate to monomer ratios were lower than for rat cardiomyoblasts. The value of the aggregate to monomer ratio for cardiac cells incubated with FCCP was lower than for the cells cultured in a culture medium. Similarly, it was observed that the ratio of two dye forms for co-culture of metabolically perturbed HCM cells with MSC cells was lower than for the cells cultured in a culture medium and slightly higher than for cells incubated with FCCP. Based on the obtained results, we can conclude that stem cells did not significantly increase the potential of the inner mitochondrial membrane. The results of the quantitative analysis of fluorescence intensity were also confirmed using microscopic observation (Figure 2C). The green fluorescence intensity corresponding to the monomers (a low mitochondrial membrane potential) increased for cardiac cells (H9C2 and HCM) maintained with FCCP. In turn, for the co-cultures, a higher intensity of orange/red fluorescence corresponding to aggregates (a high mitochondrial membrane potential) was observed. It was also noticed that stem cells aligned parallel to cardiac cells in co-cultures, especially for the co-culture with HCM cells. This may indicate that the nanofibrous mats and co-culture with cardiac cells stimulated the stem cells to parallel alignment. This is particularly important for mimicking of in vivo microenvironment where cardiac cells also aligned parallel to each other.

### 3.2. Working of the Microsystem

The geometry of the microsystem was designed in such a way that it allowed simultaneous cultures of: (1) cardiac cells under normal conditions, (2) cardiac cells under abnormal conditions, (3) cardiac cells exposed to FCCP and co-cultured with stem cells, as well as (4) cardiac and stem cells under normal conditions. The design of the Organ-on-a-Chip system was created in a CAD program (AutoCad, Autodesk) based on our previous work [34]. Unlike this work, the geometry and a size of culture microchambers were changed here. Additionally, the methods of nanofiber mats integration in the microsystem were modified. In this work, the microsystem was designed in such a way that both the microstructures and nanofiber mats were placed in the same PDMS layer. Thanks to that the repeatability of the microsystem, fabrication as well as the microsystem operation has been improved and simplified. The presented microsystem was made of two poly(dimethylsiloxane) (PDMS) (Sylgard 184, DowCorning) layers (Figure 1A). The upper layer consisted of a network of microchannels and microwells, in which nanofiber mats were placed. The nanofiber mats were integrated with a microfluidic system to ensure the parallel arrangement of cardiac cells, which better imitates the native tissue of heart muscle [34,43]. The microchannel network of the microsystem consisted of four microchannels (300 µm width, 100 µm height) with three culture microchambers (6400 µm length, 4000 µm width). The length of the nanofibers was experimentally selected to allow their reproducibly placement in culture microchambers and to ensure proper flow of fluids and cells in the microstructures. The arrangement of the microchambers enabled the analysis of three independent measurements points in the microsystem. The microchannels were connected with two inlets for introducing cell suspension (inlets 1 and 2) and with three inlets for introducing the solutions (inlets 3, 4, and 5) (Figure 1B). To carry out automatic experiments using a plate reader, the microchambers were arranged according to the wells on 96-well plate.

**Figure 3:**
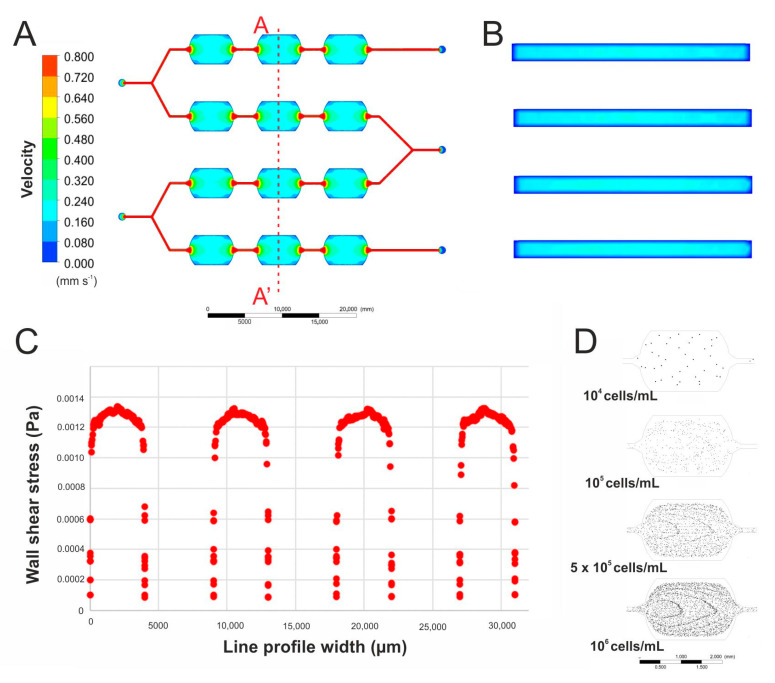
(**A**) A simulation of the culture medium flow in the designed microfluidic system. (**B**) A simulation of the culture medium flow in the culture microchambers (cross-section view). (**C**) Wall shear stress simulation in four culture microchambers. (**D**) Simulations of cell distribution after introducing a suspension of cells of different densities into the microsystem.

Due to the fabricated microsystem being a modification of our previous microdevice, new computer simulations, that enabled the assessment of the flow conditions, were performed. A flow rate of 2 μL/min at two external inlets (inlets 3 and 5) and 4 μL/min at inlet 4 were tested as flow rates that have no destructive effect on the cells. Based on the simulation of the flow in the entire microsystem, it was observed that the liquid flow velocity is the same in all microchannels and microchambers (Figure 3A). Additionally, the flow velocity distribution shown in the cross-sections of the culture microchambers is uniform over the entire volume of the microchamber (Figure 3B). A wall shear stress analysis was also carried out in each microchamber and it was approximately 0.0013 Pa (Figure 3C). Based on the achieved simulations, it can be assumed that the applied flow velocities should not negatively affect the growth of cells cultured in the designed microsystem. It was also assumed that the density of the cells introduced into the microsystem would be the same in all microchambers and cell culture conditions would be comparable. The size of the microchamber was larger in the presented system than in the microsystem previously developed. Therefore, it was simulated how the usage of different densities of cell suspension influence the number of the introduced cells in the microchambers. The simulation was performed for a flow rate of 15 μL/min (through inlets 1 and 2) and densities of cell suspension of 10^4^, 10^5^, 5 × 10^5^, and 10^6^ cells/mL (Figure 3D). The computer simulation showed that the average number of the cells in the microchamber was: 40, 403, 1666, and 2755, which gave surface densities as follow: 1.8, 18.3, 75.4, and 124.8 cells/mm^2^, consecutively for introduced cell densities of 10^4^, 10^5^, 5∙× 10^5^, and 10^6^ cells/mL. Based on these results, an experimental study was carried out in the fabricated microsystem. Experiments with the usage of a cell suspensions with the three highest concentrations were performed. In this case, the obtained surface density of the cells equaled 24.5 (for 10^5^ cells/mL), 63.9 (for 5 × 10^5^ cells/mL), and 181.9 cells/mm^2^ (for 10^6^ cells/mL) in each microchamber. Cell cultures which had been planned in the designed microsystem were performed through 96 h, therefore cell density of 5 × 10^5^ cells/mL was used in further research.

### 3.3. Evaluation of the Influence of Stem Cells on the Metabolic Activity of Cardiac Cells Incubated with FCCP

**Figure 4:**
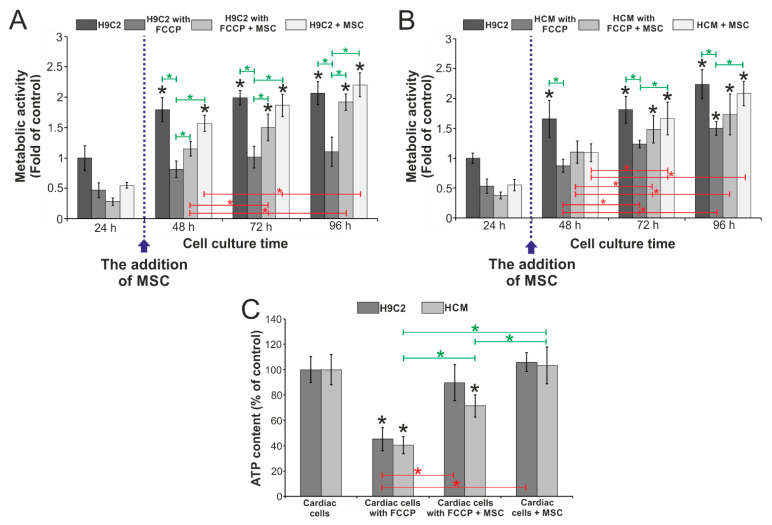
The results of metabolic activity measurements of (**A**) rat cardiomyoblasts and (**B**) human cardiomyocytes. *N* ≥ 3, asterisks (*p* < 0.05) signify statistical significance relative to control (black), between culture times for the same cell culture (red) and between different types of culture (green). (**C**) The results of measuring the adenosine triphosphate (ATP) molecule content in the four tested types of cell culture in the microsystem for two cardiac cells lines. *N* ≥ 3, asterisks (*p* < 0.05) signify statistical significance relative to control (black), between cell cultures with H9C2 (red) and between cell cultures with HCM (green).

The studies were conducted in the designed microsystem to evaluate the effect of stem cells on metabolically perturbed cardiac cells. The tests were performed simultaneously in one microsystem on four types of cell cultures: cardiac cells cultured under (1) normal and (2) abnormal conditions, cardiac cells cultured with mesenchymal stem cells under (3) normal and (4) abnormal conditions. The cell cultures were conducted in the microsystem for five days, and the daily metabolic activity of AlamarBlue was determined. The obtained results are shown in Figure 4. It was found that stem cells influence the metabolic activity of cardiac cells (H9C2 and HCM cells) incubated with FCCP. For rat cardiomyoblasts (H9C2), it was noticed that metabolic activity increased each day of the culture (Figure 4A). The effect of stem cells on metabolically perturbed H9C2 cells was observed after 48 h of cell culture in the microsystem. For 48 h culture, it was noticed that the metabolic activity of H9C2 cells incubated with FCCP and co-cultured with MSC cells was higher than the metabolic activity of H9C2 incubated with FCCP. During the next day, differences of the metabolic activity between these two types of cultures increased. On the last day, the metabolic activity was approx. 1.2 for H9C2 cells exposed to FCCP and approx. 2 for H9C2 cells (exposed to FCCP) cultured with MSC cells. Additionally, it was noticed that the metabolic activity for all types of cultures except H9C2 cells incubated with FCCP was similar on the last day. This may indicate that stem cells effect metabolic activity of cardiac cells maintained with FCCP.

Similar results were obtained for cell cultures with human cardiomyocytes (Figure 4B). After 48 h of culture, a slight increase in the metabolic activity of HCM exposed to FCCP was observed. No significant differences were found between two types of co-cultures and HCM cells incubated with FCCP. An increase in metabolic activity was observed in each of the tested cultures for 72 h, especially in the co-culture of HCM and MSC (under normal conditions). However, no significant difference was noticed between the culture of metabolically perturbed HCM cells and co-culture of these cells with MSC. On the last day of culture, an increase in metabolic activity was observed for all types of cell culture. For HCM cells incubated with FCCP, the activity equaled approx. 1.5, for co-culture of HCM (exposed to FCCP) with MSC the obtained result was slightly higher. After 96 h of cell culture, the highest metabolic activity was observed for HCM cultured in the culture medium. Contrary to the tests performed on H9C2 cells, no significant differences were observed between monoculture of HCM cells and coculture of HCM cells with MSCs both exposed to FCCP.

On the last day of cell culture in the microsystem, the adenosine triphosphate (ATP) content in the cells was also evaluated using a bioluminescence-based assay. As a result of FCCP action in cells, oxidative phosphorylation is disturbed and ATP synthesis is inhibited. Therefore, it was decided to investigate whether the presence of stem cells would affect the efficiency of ATP synthesis in the tested cell culture. The aim of this test was to determine the condition of the cell cultures after 96 h and to evaluate the influence of MSC cells in the functioning of mitochondria. A cardiac cell culture in a medium was chosen as a control. The results of the experiment are presented in Figure 4C. For the cells incubated with FCCP, the content of ATP molecules was about 40% compared to the control. Higher values were obtained for cardiac cells incubated with FCCP and co-cultured with stem cells. For the co-culture H9C2 cells (maintained with medium) with MSC cells, the ATP content was approx. 90% and for HCM cells with MCS cells it was approx. 71%. While the results obtained for co-cultures of both cardiac cell lines with stem cells cultured in a medium were slightly higher than the control. Significant differences were observed between the types of cultures for both cardiac cell lines. Higher ATP content in the co-cultures metabolically perturbed cardiac cells with stem cells may be caused by the addition to the culture of stem cells in which ATP synthesis was not inhibited by the action of FCCP agent. Therefore, it was found that mesenchymal stem cells do not significantly affect the mitochondrial function in cardiac cells incubated with FCCP.

## 4. Discussion

Evaluation of the effect of stem cells on pathological, especially hypoxic cardiac cells is particularly important in conducting research on cell therapy in people suffering from ischemic heart disease. Because heart transplantation is the most effective treatment and the number of donors is limited, scientists conduct intensive research on the use of stem cells to regenerate damaged heart muscle. Many regeneration strategies that combine the use of cell, soluble factors, biomaterials, and their combination are being developed. To carry out reliable research, it is necessary to use appropriate cell models that would mimic in vivo conditions as well as possible [44]. Because of their numerous advantages, Organ-on-a-Chip microsystems enable the development of such models [21,23,45].

The research was carried out using two lines of heart muscle cells (H9C2 and HCM) and human mesenchymal stem cell (MSC). MSCs are often used in organ regeneration studies because they are easy to isolate. In addition, they secrete biomolecules that activate the tissue repair process. MSCs are capable of differentiation into cardiomyocytes under appropriate culture conditions [46]. Consequently, MSC cells are often used in research into their regenerative effects on damaged cardiac tissue. There are examples in the literature that describe the advantages of using a co-culture of stem cells with other cell types. Mummery et al. used a co-culture of embryonic stem cells (ESC) with visceral endoderm-like cells (END-2). It was confirmed that the presence of END-2 cells stimulated the ESCs for differentiation into cardiac cells which exhibited spontaneous beating [30]. In other studies, human MSCs and rat cardiomyocytes were used. It was confirmed that intercellular junctions are formed between the cells of the two lines. Transfer of cytosol and mitochondria from MSCs to cardiomyocytes through the formed junctions was also noticed. Moreover, because of the presence of cardiomyocytes, it was possible to observe the differentiation of stem cells into cardiac cells [31]. In turn, Gao et al. studied co-culture amniotic fluid-derived stem cells with human cardiomyocytes. The presence of cardiac cells increased the expression of troponin T (a protein characteristic of cardiomyocytes) in stem cells. However, these cells did not exhibit the functional and morphological characteristics of mature cardiomyocytes. In this case, the presence of cardiomyocytes was not a sufficient factor to cause complete differentiation of stem cells [18]. Researchers focused mainly on the effect of cardiac cells on the differentiation of stem cells into cardiomyocytes in all the described examples. However, there are a few examples of studies that describe how stem cells affect the functioning of cardiac cells. Contrary to literature reports, studies on the effect of stem cells on cardiac cells were presented in this paper. Preliminary macroscale research confirmed that MSCs improved the metabolic activity of cardiac cells incubated with 10 µM of FCCP.

Microfluidic systems have advantages over standard in vitro studies. Mainly, because microsystems allow culture conditions similar to in vivo to be obtained. Therefore, based on our previous work it was decided to develop a microfluidic system integrated with nanofiber mats, in which it was possible to simultaneously conduct independent cell cultures. The presented microsystem has optimized the geometry and nanofiber mats integration. Because the structure of nanofibers resembles ECM and they stimulate parallel growth of the cells, the developed cellular model more closely mimicked cardiac tissue than conventional monolayer models. In the developed microsystem, cardiac cells (H9C2 or HCM) were treated with a biochemical factor simulating the state of abnormal conditions. Next, the effect of mesenchymal stem cells on cardiac cells growth was assessed. Ma et al. also used MSC cells to regenerate damaged cardiac tissue in the microfluidic system. The presented microsystem consisted of a PDMS membrane that was connected to an array of microelectrodes. The PDMS membrane consisted of eight microchannels in which rat cardiomyocytes were cultured. Then, some of the cardiac cells were removed, creating a gap in which rat MSCs were placed. The added stem cells were used to connect the separated parts of the heart muscle. The newly formed connection exhibited the ability to conduct an electrical signal along the entire fiber (made of the cells) [47]. H9C2 cells were also cultured in a microsystem under hypoxic conditions provided using FCCP. Next, skeletal myoblasts (L6) were investigated to study regeneration of hypoxic H9C2 cells [38]. In the cited works, the scientists proved that it is possible to model heart failure in the microfluidic system. Moreover, there are not many works in which the influence of stem cells and myoblasts on the improvement of the function and metabolic activity of cardiac cells have been studied. The described studies were carried out on cells cultured in the form of monolayers, which does not fully reflect in vivo conditions. In the research presented in this paper, nanofibers were used which were integrated with the designed microfluidic system used to study four different cultures at the same time. The research was also carried out on human cardiomyocytes (HCM) which have not been used in experiments concerning myocardial disfunction study so far. Based on the obtained results, it was found that the metabolic activity was increased in cardiac cells incubated with FCCP and co-cultured with stem cells compared to the culture of cells incubated with FCCP and co-culture of cardiac cells with stem cells cultured with a culture medium. Therefore, it was proved that stem cells improved the metabolic activity of cardiac cells which were metabolically perturbed.

## 5. Conclusions

To conclude, the study of stem cells’ influence on the metabolic activity of cardiac cells incubated with FCCP was performed under microfluidic conditions. The developed microsystem allows four different cell cultures under different conditions to be obtained: (1) cardiac cells under normal conditions, (2) cardiac cells under abnormal conditions, (3) cardiac cells exposed to FCCP and co-cultured with stem cells, as well as (4) cardiac and stem cells under normal conditions. Additionally, the integration nanofibers (PLLA and PU) with the microchambers mimic in vivo conditions more than standard well-plate or microsystems without additional biomaterials. The obtained results confirmed that the co-culture of cardiac cells exposed to FCCP with stem cells influenced the condition of the cell culture. It was proved that the metabolic activity of cardiac cells incubated with FCCP and co-cultured with stem cells was increased. Based on the obtained results, it was found that the synthesis of ATP molecules was higher for co-culture of metabolically perturbed cardiac cells with stem cells compared to the monoculture of cardiac cells maintained with FCCP. Thus, it was proved that stem cells improve the metabolic activity of cardiac cells incubated with FCCP. Additionally, the fabricated microsystem allows the usage and study of the influence of a few differentiation factors at the same time (biochemical factors, the influence of the cell culture surface or the selection of appropriate shear stress values). Therefore, it can be useful in further study of heart cell regeneration.

## Data Availability

Not applicable.
